# SARS-CoV-2, CT-Values, and Infectivity—Conclusions to Be Drawn from Side Observations

**DOI:** 10.3390/v13081459

**Published:** 2021-07-27

**Authors:** Martin Platten, Dennis Hoffmann, Roger Grosser, Fabian Wisplinghoff, Hilmar Wisplinghoff, Gerhard Wiesmüller, Oliver Schildgen, Verena Schildgen

**Affiliations:** 1 Labor Dr. Wisplinghoff, Horbeller Str. 20, D-50858 Köln, Germany; m.platten@wisplinghoff.de (M.P.); d.hoffmann@wisplinghoff.de (D.H.); r.grosser@wisplinghoff.de (R.G.); f.wisplinghoff@wisplinghoff.de (F.W.); h.wisplinghoff@wisplinghoff.de (H.W.); 2 Institut für Virologie und Mikrobiologie, Universität Witten/Herdecke, Stockumer Str. 10, D-58453 Witten, Germany; 3 Gesundheitsamt der Stadt Köln, Infektions- und Umwelthygiene, Neumarkt 15-21, D-50667 Köln, Germany; gerharda.wiesmueller@stadt-koeln.de; 4 Institut für Pathologie, Klinikum der Privaten Universität Witten/Herdecke, Ostmerheimer Str. 200, D-51109 Köln, Germany

In their recent article published in Viruses, Michel Drancourt and colleagues [1] have made an interesting but underestimated side-observation. As shown in Figure 1, they were able to isolate infectious SARS-CoV-2 virus from a clinical sample with a low concentration of viral RNA, which is reflected by a PCR Ct-value of 33. This finding is of foremost importance, because many current hygiene concepts rely on the statistical assumption that the likelihood of infectivity decreases reciprocally to the increase of the Ct-value. While this is true under certain circumstances, the conclusion that patients with high Ct-values are no longer able to transmit infectious virus particles adversely affects a successful pandemic management as long as no minimal infectious dose is reliably determined. Furthermore, it has to be considered that many discharge management concepts are still based on data already obtained from early on in the pandemic, including time-lines of infection, Ct-value threshold >30, or copy number threshold >10^6^/mL [2,3].

Among the early publications implying that patients with Ct-values ≥ 30 are not infectious anymore is another study by the group of Didier Raoult [4], which already has unintendedly shown that 50% of clinical specimens with Ct-values ≥ 30 can be cultured and therefore may be potentially infectious.

For this reason, it remains an important issue to reduce person-to-person transmission of SARS-CoV-2 on an individual, as well as on a public, health level in order to stop the pandemic. Currently, besides rapid and comprehensive vaccination of the population, different diagnostic strategies have been demanded by politicians and health care experts to reach this objective. As one strategy, rapid antigen tests (RAT) have been performed either by health professionals or through self-testing to identify SARS-CoV-2-positive individuals in vulnerable institutions such as nursing homes, despite a controversial discussion regarding their diagnostic reliability [5,6]. Although antigen tests can be of use for the rapid identification of highly infectious individuals [7,8], it is important to keep in mind that these tests are primarily intended and best-suited for use in symptomatic patients and have a markedly reduced sensitivity for detection of SARS-CoV-2 in comparison with PCR in samples containing lower viral loads [9,10].

In general, it is attempted to define viral load thresholds above which infectivity of COVID-19 patients is assumed. In daily practice, PCR Ct-values are used as surrogate markers for the amount of virus in a given sample and more importantly, to deduce the patient’s infectivity. However, it is not yet known how many SARS-CoV-2 virions are required to cause an infection, how long infective virus persists in patients through different stages of infection, or whether Ct-values correlate with the number of infectious virus particles. The fact that an absolute quantification of viral load is impossible due to specimen quality and nature and inter-laboratory comparability is aggravated due to a variety of methods, instruments, and different combinations which impede a proper evaluation of the pandemic situation. Buchta et al. addresses this issue, in particular regarding the limitations of interpreting Ct-values with respect to different SARS-CoV-2 target genes when different combinations of extraction platforms/reagents and RT PCR platforms/reagents were used [3]. When they analyzed the outcome of an external quality assessment challenge, it turned out that quantitative results deviate in 7.7% of cases by more than ±4 cycles (up to 18 cycles) from the respective individual means, leading to the conclusion that standardization is needed, if patient management procedures should be based on SARS-CoV-2 (RT) PCR Ct-values.

As mentioned above, many correlations and assumptions are based on research published from early on in the pandemic, including a meta-analysis of Cevik et al. [11] Due to its early publication and the fact that the study covers publications exclusively until the end of June 2020, some issues could only be discussed preliminarily, especially as SARS-CoV-2 is the most rapidly growing medical and scientific field, with 44,033 new entries in PubMed between the 1 July 2020 and the 31 December 2020, with a mean of 7338 publications per month, including several papers reporting long-lasting shedding of viable virus in the course of SARS-CoV-2 infection [6,7,8,9,10]. In contrast, Cevik et al. summarized that shedding of viral particles able to cause cytopathic effects in cell cultures is not possible later than 9 days after symptom onset, although the authors of at least one underlying original publication admitted that cultivation of SARS-CoV-2 later than 9 days after symptom onset was not attempted [12].

While the studies taken into account by Cevik et al. were highly important reports and triggered subsequent COVID-19 research, many of them were preliminary in the sense that the development of culturing systems for SARS-CoV-2 has progressed further since then and more sensitive methods for virus isolation are now available [13].

In this context Brandolini et al. have shown that in a range of 10^2^ to 10^6^ SARS-CoV-2 RNA copies per µL (≙ 10^5^ to 10^9^ copies/mL), the Ct-values range between 32 and 17. These Ct-values correspond to approximately 10^2^ to 10^5^ TCID 50. This means that Ct-values between 17 and 32 represent culturable virus amounts and thus have to be assumed to be infectious (14). Against this background, the study by Wölfel and colleagues [14] underestimated their data with respect to infectivity when they claimed that no culturing of SARS-CoV-2 was possible either because the RNA amount was below 10^6^ copies, performed more than 8 days after symptom onset or when prepared from stool. These conclusions have now been refuted by Zhou et al. [15], who have shown that organoid cell cultures may support viral replication also from stool samples. They were able to isolate SARS-CoV-2 from a stool specimen of a 68-year-old woman who tested positive for the virus at a Ct-value of 33.6, which in light of Bardolini et al. corresponds to a copy number below 10^5^ copies per ml, suggesting that previously used culturing systems seem to be not permissive enough for SARS-CoV-2 culture from stool.

Additional studies have also shown that culturing of SARS-CoV-2 is possible with samples containing significantly less than the previously claimed culturing threshold of 10^6^ genome equivalents [13] and that successful cultivation after day 8 from sampling or symptom onset is also possible [16,17]. Beyond this, a recent study from Switzerland demonstrated that a number of further environmental factors, such as air–liquid interface, contact and temperature difference, are further important factors for successful SARS-CoV-2 replication in cell culture, leading to the conclusion that we still cannot rely on Ct-values as a marker for infectiousness [18].

The debate on the period of infectivity is closely linked to the discussion on PCR Ct-values. As a higher Ct-value represents a lower amount of viral RNA in a given sample, it is often used as a surrogate parameter for the amount of infectious particles and hence infectivity, but the relevance of any Ct-value threshold as a measure of infectivity remains unclear. The study of Aron et al. showed that 2 out of 8 samples (25%) with Ct-values higher than 30 could still be successfully cultured (see Figure 2 of [19]). Although the likelihood of culturing success decreases to 6%, Singanayagam and coworkers have shown that culturable virus may be shed more than 10 days after onset of symptoms, despite Ct-values > 35 [20] leading to infection control difficulties in periods of high infection rates, as observed in India, as it cannot be excluded that in rare cases patients can shed a viable virus more than 10 days after symptom onset.

In addition, Kujawski and coworkers were also able to isolate a culturable virus from samples with higher Ct-values (>30) [12], suggesting that theoretically even small amounts of infectious particles might be enough to initiate an infection in vivo as long as no reliable data on the necessary minimum infective dose exists. This is especially important with regard to the novel virus variants of concern B1.1.7 (alpha; UK), B1.135 (beta; South Africa), and P.1 (gamma; Brazil), which have been shown to be more transmissible in recent studies [21,22,23,24,25,26,27,28,29], as well as for the delta variant for which not enough peer reviewed data regarding the correlation of infectivity and Ct-values are yet available.

The use of RATs is increasingly popular and some countries have discussed the easing of pandemic-related restrictions or exemptions from hygiene measures based on negative test results, but it must be emphasized that there is no data supporting the conclusion that an individual with a negative rapid antigen test cannot be infectious. As there is no definitive Ct-value threshold beyond which antigen tests consistently yield false-negative results, we compared the results of SARS-CoV-2 PCR (Allplex SARS-CoV-2, Seegene, Düsseldorf, Germany), a laboratory SARS-CoV-2-Ag test (Vitros^®^ 3600 Immunodiagnostic System, Ortho Clinical Diagnostics, Raritan, NJ, USA) as well as three rapid SARS-CoV-2-Ag tests (Panbio™ COVID-19 Ag Rapid Test Device, Abbott, Cologne, Germany; SARS-CoV-2 Rapid Antigen Test, SD Biosensor/Roche, Mannheim, Germany; SARS-CoV-2 Antigen Rapid Test, Lepu Medical, Beijing, China) to evaluate a Ct-value threshold below which rapid antigen tests match reliably with positive PCR results. In our setting, the analysis of 451 quality control data samples suggest that RATs are frequently negative in PCR-positive samples with Ct-values above 24–28. This matches the findings of other studies including one performed at a large German maximum care hospital with 468 samples that identified Ct-values ≤ 22 as the limit for a 100% correlation of PCR and rapid antigen test [30].

To estimate the potential consequences of using RATs instead of PCR, irrespective of their intended use in symptomatic patients, a number of 1,259,559 respiratory samples (median age: 45.7 years (range 0–101, SD21.9); 52.4% female and 47.6% male patients) obtained during clinical routine diagnostic from 1 September to 23 December 2020 was analyzed. Of these, 64,903 samples (5.2%) were PCR-positive and subjected to Ct-value scattering. Based on PCR CT values between 24–28 as the respective detection limit, antigen tests could have missed up to 43,204–35,409 (64.2–52.6%) of SARS-CoV-2 infections which tested positive by PCR (Figure 1). These data do not allow any general estimation of sensitivity or specificity of antigen tests per se, but it clearly shows that the use of RATs could lead to a profound percentage of undetected SARS-CoV-2 infections, especially in asymptomatic individuals. This is of relevance as it is already known that asymptomatic patients can also spread the virus and SARS-CoV-2 infections are not reliably identified by rapid antigen testing in these individuals [5,6,31,32,33,34,35,36,37,38,39]. Taking into account that it is postulated that there are between 2.6- and 8-fold more SARS-CoV-2 infections than those identified by testing [40,41], either because they are asymptomatic or because their symptoms are too mild to initiate testing algorithms, false negative antigen tests could boost an unnoticed and uncontrolled viral spread due to an unjustified sense of security.

A total of *n* = 1,259,559 specimens was tested by PCR, of which *n* = 64,903 tested SARS-CoV-2 positive. The Ct-value scattering of the PCR positive specimens is shown in the graph. Arrows exemplarily indicate to what percentage Ct-values of PCR and rapid antigen test (RATs) results did not correlate. This diagnostic gap could have led to up to 35,409 (52.6%)–43,204 (64.2%) missed SARS-CoV-2 infections if RATs were used as the sole diagnostic tool.

Even though numerous publications, including the study of Drancourt et al., showed that culturable SARS-CoV-2 can be isolated under suboptimal conditions, no minimal infectious dose has yet been determined, although a reliable correlation with viral load and rapid test positivity should be achieved. For this reason, the use of RATs in asymptomatic individuals, contrary to their original intended use, is only justified by the fact that in terms of public health, special attention should be given to the probability of infection establishment, which is assumed to correlate with viral load. However, the probability of infection is also dependent on exposure time, the (so far unknown) infectious dose, as well as prolonged virus shedding [42]. Moreover, transmissions are more probable if infected individuals tend to have an increased production of saliva or a higher droplet load [43]. This means that there is a possibility of transmission, although, according to widespread assumptions, a low viral load in the upper respiratory tract excludes an ability of infection establishment. Therefore, it should be considered that negative RAT results may change the test person’s behavior, leading to a sloppier handling of hygiene rules and pandemic restrictions. As it was shown that advanced cell cultures seem to be more susceptible for infection and that environmental factors influence viral replication, future attention should be paid to the development of cell culture systems, which enable standardized reliable cultivation of SARS-CoV-2 to identify determinants influencing infectiousness. This is important, because individual variation in infectiousness should be considered to understand emerging disease outbreaks, especially with regard to “superspreading events” [44]. Therefore, from a public health perspective, Ct-value cut-offs can be defined as acceptable low risk value, not more, not less.

## Figures and Tables

**Figure 1:**
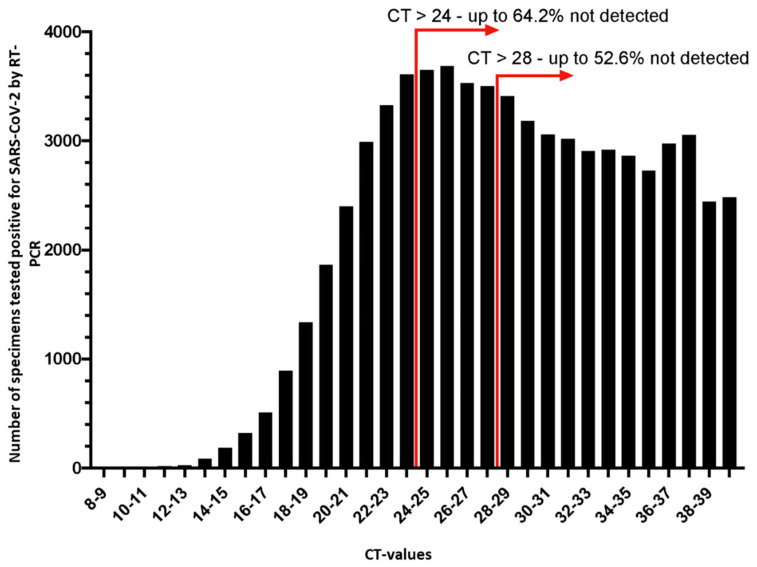
SARS-CoV-2-PCR Ct-value scattering of respiratory samples collected between 1 September and 23 December (2020).
